# MiR-199a/b-3p inhibits gastric cancer cell proliferation via down-regulating PAK4/MEK/ERK signaling pathway

**DOI:** 10.1186/s12885-017-3949-2

**Published:** 2018-01-05

**Authors:** Bin Zeng, Wei Shi, Gao Tan

**Affiliations:** 1 Department of Gastroenterology, The First Affiliated Hospital of South China University, Hengyang, 421001 China; 2 Department of Gastroenterology, The People’s Hospital of Yangxin County, Yangxin, 435200 China; 3 Guangdong Provincial Key Laboratory of Gastroenterology, Department of Gastroenterology, Nanfang Hospital, Southern Medical University, 1838 N. Guangzhou Ave, Guangzhou, 510515 China

**Keywords:** MiR-199a/b-3p, Gastric cancer, PAK4, ERK

## Abstract

**Background:**

Gastric cancer (GC) is one of the most frequent malignant tumors and the molecular mechanism underlying its proliferation remains far from completely understood. Although accumulating evidence shows that abnormal expression of microRNA (miRNA) is involved in tumorigenesis, the role of specific miRNAs involved in GC remains elusive. MiR-199a/b-3p functions as a tumor suppressor in diverse cancers, but its expression, function, and mechanism in GC remain unclear. Our aim is to explore miR-199a/b-3p expression and its role in regulating GC cell proliferation.

**Methods:**

Real-time PCR was performed to determine miR-199a/b-3p expression in GC tissues and normal adjacent tissues as well as normal gastric mucosal cell line GES-1 and GC cell lines MGC-803 and SGC-7901. MTT assay and Western blot were performed to determine cell proliferation and expression of PAK4, p-MEK and p-ERK, respectively. MiR-199a/b-3p mimics-transfected assay and PAK-specific siRNA assay were performed to determine their function in cell proliferation, respectively. GC xenograft nude mice were used to determine miR-199a/b-3p function in cell proliferation.

**Results:**

MiR-199a/b-3p expression was significantly decreased in GC tissues and GC cell lines MGC-803 and SGC-7901. MiR-199a/b-3p over-expression and PAK4 silencing inhibited cell proliferation and diminished the activation of p-MEK and p-ERK in MGC-803 and SGC-7901 cells, and miR-199a/b-3p over-expression reduced PAK4 expression. MiR-199a/b-3p over-expression suppressed MGC-803 cell growth and PAK4 expression in nude mice.

**Conclusions:**

miR-199a/b-3p inhibits GC cell proliferation via down-regulating PAK4/MEK/ERK signaling pathway and may be a novel prognostic biomarker and a potential therapeutic target for GC patients.

**Electronic supplementary material:**

The online version of this article (10.1186/s12885-017-3949-2) contains supplementary material, which is available to authorized users.

## Background

Gastric cancer (GC) is the second leading cause of cancer-related deaths and the fourth most frequent malignant tumors worldwide [1]. Along with the elevation of its incidence rate, it has become one of the major threatens to human health. As most human cancers, GC pathogenesis is associated with multiple factors involving activation of proto-oncogenes and/or inactivation of tumor-suppressor genes. However, the molecular mechanism underlying its proliferation remains far from completely understood.

MiRNAs are small non-coding RNA molecules with approximately 20–25 nucleotides in length and play important roles in the pathogenesis of many human diseases through modulating their specific target activity [2, 3]. Although accumulating evidence shows that abnormal miRNA expression is involved in tumorigenesis [4, 5], the role of specific miRNAs involved in GC remains elusive. MiR-199 was first identified in 2003 [6]. Its two putative hairpin precursors in human map to chromosome 19 and chromosome 1, and the mature forms of its excised miR sequences are named miR-199a/b-5p and miR-199a/b-3p [7, 8]. Recent studies showed that miR-199a/b-3p function as a tumor-suppressor gene and inhibits cell proliferation in some cancers, such as human hepatocellular carcinoma [7], papillary thyroid carcinoma [9], endometrioid adenocarcinoma [10], renal cell carcinoma [11], osteosarcoma [12], ovarian cancer [13] and breast cancer [14]. However, its expression, function and potential mechanism in GC remain unclear.

The purpose of this study is to investigate miR-199a/b-3p expression and its role in regulating GC cell proliferation. We found that miR-199a/b-3p expression was decreased in GC tissues and cell lines, and identified it as a critical suppressor of GC cell proliferation in both in vitro and in vivo studies. We also showed that miR-199a/b-3p functions as a GC suppressor via down-regulating PAK4/MEK/ERK signaling pathway.

## Methods

### Clinical samples

Twenty fresh GC tissue samples from GC patients and their matched adjacent normal gastric mucosal tissues were immediately snap frozen in liquid nitrogen and stored at −80 °C until total RNA was extracted. The samples were collected from consenting individuals according to the protocols approved by the Ethics Committee at First Affiliated Hospital of South China University. The association of miR-199a/b-3p relative expression with the clinicopathological characteristics in 20 GC patients are showed in Additional file 1: Table S1.

### Cell culture

Human normal gastric mucosa cell line GES-1 (CBP60512) and human gastric cancer cell lines MGC-803 (CBP60485) and SGC-7901 (TCHu 46) were all purchased from Nanjing Cobioer Biotechnology and Shanghai Cell Bank of Chinese Academy of Sciences, and was cultured in RPMI 1640 medium (HyClone) supplemented with 10% fetal calf serum (HyClone), 2 mM L-glutamine, 100 U/ml Penicillin, and 100 mg/ml streptomycin at 37 °C in a humidified atmosphere with 5% CO2. The cells were used between passages 10 and 20. The complete medium was refreshed every 24 h.

#### In vivo tumor xenograft studies

Four-week-old male athymic BABL/c nude mice were used under conditions approved by the Institutional Animal Care and Use Committee of First Affiliated Hospital of South China University. To assess the anti-proliferation capacity of miR-199a/b-3p in vivo, 2 × 10^6^ MGC-803 cells transfected with negative control (NC) or 50 nM miR-199a/b-3p mimics respectively, were suspended in 0.2 ml sterile saline and subsequently implanted subcutaneously into the axillary fossae of each mouse. After 2 week implantation, mice were killed, and xenograft tumors were removed intactly. The volume of xenograft tumors was calculated as follows: length × width^2^ × 1/2. And fresh tissues from xenograft tumors were immediately snap frozen in liquid nitrogen and stored at −80 °C until total RNA and protein were extracted.

### Oligonucleotides and transfection

MiR-199a/b-3p mimics and negative control (NC) were chemically synthesized by RIBOBIO and transfected with Lipofectamine 2000 (Invitrogen) in MGC-803 and SGC-7901 cells at a final concentration of 10 or 50 nM. PAK4-spedific siRNA and transfection reagent were purchased from Santa Cruz Biotechnology. Both MGC-803 and SGC-7901 cells were transfected with transfection reagent only (Mock) or the mixture of transfection reagent and 50 nM PAK4 siRNA according to the manufacturer’s protocol.

### Real-time quantitative RT-PCR

Total RNA was extracted from the prepared cells and tissues using the RNAiso Plus Kit (Takara), then cDNA synthesis was performed using the PrimeScript RT reagent Kit (Takara), and finally Real-time PCR was performed in triplicate using the LightCycler 480 System (Roche) according to the manufacturer’s protocol. The PCR primers were shown in Additional file 1: Table S2. U6 and β-actin were used as internal controls. The 2^−ΔΔCT^ method was used to analyze real-time PCR data.

### Western blot

The prepared cells and tissues were lysed for 30 min on ice in RIPA lysis buffer (10 mM Tris (pH 8.0), 150 mM NaCl, 1% Nonidet P-40, 0.1% SDS, and 0.5% deoxycholate, supplemented with the protease inhibitor PMSF. After being centrifuged at 14,000×g for 30 min at 4 °C the supernatants were collected. SDS-polyacrylamide gelelectrophoresis and western blot were performed according to standard protocols. Human and mouse monoclonal antibodies of anti-PAK4, anti-p-MEK, anti-p-ERK and anti-β-actin (Cell Signaling Technology) were all diluted at 1:1000. Secondary antibodies were all diluted at 1:4000.

### Cell proliferation assay

Cell proliferation was assessed using MTT (3-(4,5-dimethylthiazol-2-yl)-2,5-.

diphenyltetrazolium bromide, Sigma) assay. In brief, the prepared cells were seeded into 96-well plates, of which 20 μl MTT (5 mg/ml) was added into each well, and subsequently the plates were incubated in 5% CO2 at 37 °C incubator. After 4 h, the liquid was thrown away and 150 μl dimethyl sulfoxide (Sigma) was added into each well. After vibrating 10 min, the optical density was measured in a microplate reader at the wave length of 570 nm.

### Statistical analysis

The results are shown as the mean ± standard deviation. Statistical significance was determined by one-way analysis of variance with Tukey’s multiple comparisons under equal variances or with Dunnett T3’s multiple comparisons under unequal variances; Statistical significance of gene expression between normal and cancerous tissues was determined by Pearson chi-square test; a value of *P* < 0.05 was considered statistically significant.

## Results

### MiR-199a/b-3p inhibits GC cell proliferation in vitro

**Fig. 1:**
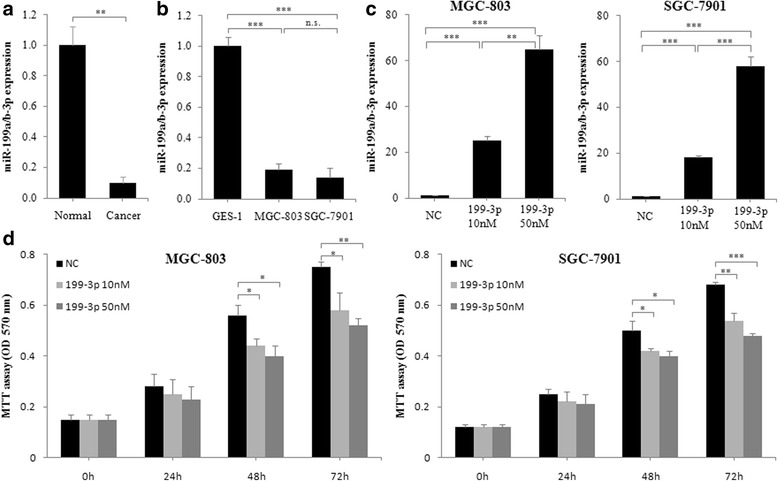
miR-199a/b-3p inhibits GC cell proliferation in vitro. **a** GC tissues and the matched adjacent normal gastric mucosal tissues were collected from 20 patients. Their miR-199a/b-3p expression was detected using real-time PCR. **b** MiR-199a/b-3p expression in a normal gastric mucosa cell line GES-1, GC cell lines MGC-803 and SGC-7901 was detected using real-time PCR. **c** MGC-803 and SGC-7901 cells were transfected with negative control (NC) or miR-199a/b-3p mimics at a final concentration of 10 or 50 nM, at 48 h post-transfection, miR-199a/b-3p expression was detected using real-time PCR. **d** MGC-803 and SGC-7901 cells were transfected as in (**c**), then cell proliferation levels were detected using MTT assay at 0, 24, 48 and 72 h post-transfection. **a** Data are shown as the mean ± SD (*n* = 20). **b**-**d** Data are shown as the mean ± SD of three independent experiments. n.s., not significant. *P < 0.05; **P < 0.01; ***P < 0.001

In order to investigate miR-199a/b-3p expression in GC, we compared the expression levels of miR-199a/b-3p between the GC tissues and the normal adjacent tissues by real-time PCR. We found that the expression levels of miR-199a/b-3p were significantly lower in the cancer tissues than in the normal tissues (Fig. 1a). In addition, Compared with the normal gastric mucosal cell line GES-1, the GC cell lines MGC-803 and SGC-7901 had lower expression levels of miR-199a/b-3p (Fig. 1b).

To determine the role of miR-199a/b-3p in regulating GC cell proliferation, we assessed the cell viability of MGC-803 and SGC-7901 cells transfected with miR-199a/b-3p mimics or negative control at 24, 48 and 72 h post-transfection by MTT assay. We found that MGC-803 and SGC-7901 cells transfected with miR-199a/b-3p mimics had significantly increased expression levels of miR-199a/b-3p in a dose-dependent way (Fig. 1c), while had significantly decreased viable cell levels at 48 and 72 h post-transfection as compared with cells transfected with negative control (Fig. 1d). Similarly, SGC-7901 cells transfected with miR-199a/b-3p mimics had significantly decreased viable cell levels at 48, 72 and 96 h post-transfection by CCK8 assay (Additional file 1: Figure S2A). These results indicate that miR-199a/b-3p can inhibit GC cell proliferation in a dose-dependent way.

### MiR-199a/b-3p down-regulates PAK4 expression in vitro

**Fig. 2:**
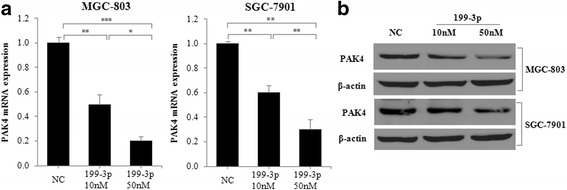
miR-199a/b-3p down-regulates PAK4 expression in vitro***.*** **a, b** MGC-803 and SGC-7901 cells were transfected with negative control (NC) or miR-199a/b-3p mimics at a final concentration of 10 or 50 nM, at 48 h post-transfection, PAK4 mRNA and protein expression was detected using real-time PCR and western blot, respectively. Data are shown as the mean ± SD of three independent experiments. *P < 0.05; **P < 0.01; ***P < 0.001

Because miRNAs function mainly through suppressing their target genes, we further predicted and analyzed the target genes of miR-199a/b-3p that functions in GC pathogenesis by TargetScan. In hundreds of its predicted target genes (http://www.targetscan.org), PAK4 not only contains two putative conserved target sites of miR-199a/b-3p (Additional file 1: Table S3), but also is over-expressed [15] and have an oncogenic role in GC [16]. Therefore, we reasoned that miR-199a/b-3p inhibits GC cell proliferation may through down-regulating PAK4 expression. To address this issues, we transfected MGC-803 and SGC-7901 cells with miR-199a/b-3p mimics or negative control, and compared their expression levels of PAK4 mRNA and protein. We found that MGC-803 and SGC-7901 cells transfected with miR-199a/b-3p mimics had significantly decreased expression levels of PAK4 mRNA and protein in a dose-dependent way as compared with cells transfected with negative control (Fig. 2a and b).

**Fig. 3:**
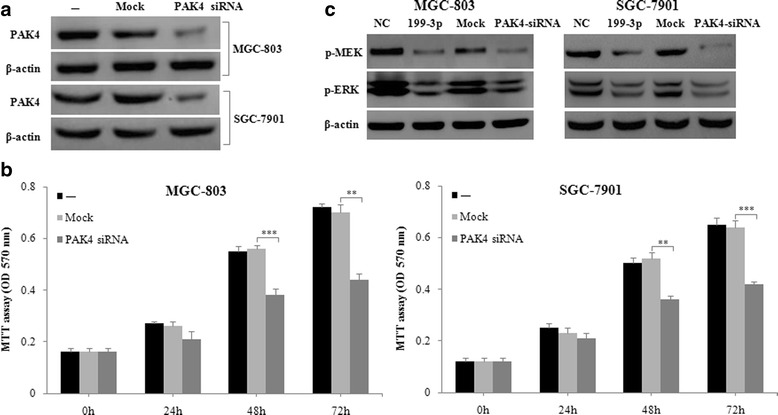
PAK4 silencing inhibits GC cell proliferation via MEK/ERK pathway in vitro. **a** MGC-803 and SGC-7901 cells were transfected with transfection reagent only (Mock) or PAK4 siRNA (50 nM), at 48 h post-transfection, protein expression of PAK4 and β-actin (internal control) was detected using western blot. **b** MGC-803 and SGC-7901 cells were transfected as in (**a**), then cell proliferation levels were detected using MTT assay at 0, 24, 48 and 72 h post-transfection. **c** MGC-803 and SGC-7901 cells were transfected with negative control (NC), miR-199a/b-3p mimics (50 nM), transfection reagent only (Mock) or PAK4 siRNA (50 nM), at 48 h post-transfection, protein expression of p-MEK, p-ERK and β-actin (internal control) was detected using western blot Data are shown as the mean ± SD of three independent experiments. **P < 0.01; ***P < 0.001.

To further determine PAK4 function in GC proliferation, we transfected MGC-803 and SGC-7901 cells with PAK4 siRNA or transfection reagent only (Mock), and assessed their cell viability by MTT assay. We found that MGC-803 and SGC-7901 cells transfected with PAK4 siRNA had lower protein levels of PAK4 and lower viable cell levels at 48 and 72 h post-transfection as compared with Mock-transfected cells (Fig. 3a and b). Similarly, SGC-7901 cells transfected with PAK4 siRNA had significantly decreased viable cell levels at 48, 72 and 96 h post-transfection by CCK8 assay (Additional file 1: Figure S2A). This results indicate that PAK4 silencing can inhibit GC cell proliferation.

### MiR-199a/b-3p suppresses PAK4/MEK/ERK pathway in GC in vitro

We subsequently performed enrichment analysis of cell signaling pathways using Gene Cloud of Biotechnology Information (GCBI) pathway database (https://www.gcbi.com.cn) to identify signaling pathways enriched by hundreds of predicted targets of miR-199a/b-3p. We found that the most enriched was MAPK pathway (Additional file 1: Figure S1). Moreover, in the MAPK pathway, the MEK/ERK pathway plays a critical role in PAK4-induced regulation of cell proliferation [17]. Therefore, we investigate whether the MEK/ERK pathway was involved in miR-199a/b-3p antitumor effect by targeting PAK4 in GC. To explore it, we transfected MGC-803 and SGC-7901 cells with negative control (NC), miR-199a/b-3p mimics, transfection reagent only (Mock) or PAK4 siRNA, and assessed their protein expression by western blot. We found that the protein activation levels of p-MEK and p-ERK were decreased in MGC-803 and SGC-7901 cells transfected with miR-199a/b-3p mimics (PAK4 siRNA) as compared with cells transfected with NC (Mock) (Fig. 3c).

### MiR-199a/b-3p inhibits GC proliferation by decreasing PAK4 expression in vivo

**Fig. 4:**
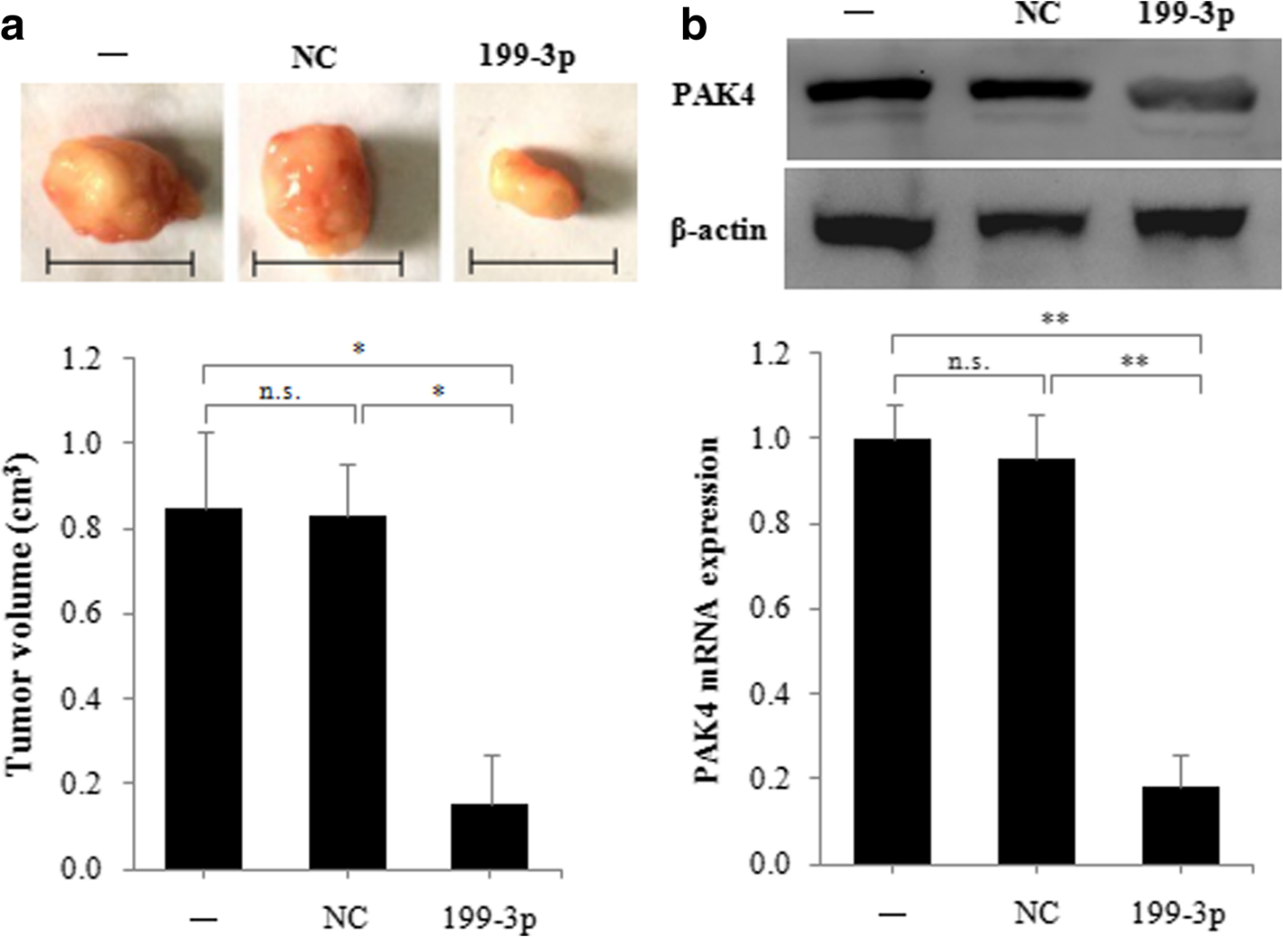
miR-199a/b-3p suppresses gastric cancer cell proliferation via down-regulating PAK4 expression in vivo*.* **a** MGC-803 cells transfected with negative control (NC) or 50 nM miR-199a/b-3p mimics respectively, were implanted into the axillary fossae of each mouse, after 2 weeks, xenograft tumors were removed intactly. Top, representative tumor images; the bar represents 1 cm. Bottom, quantitative analysis of tumor volume. **b** Total RNA and protein were isolated from xenograft tumors, and expression of PAK4 mRNA (bottom) and protein (top) was detected using real-time PCR and western blot, respectively Data are shown as the mean ± SD (*n* = 8). n.s., not significant. *P < 0.05; **P < 0.01.

To further confirm that miR-199a/b-3p suppresses GC cell proliferation. We transfected MGC-803 cells with negative control (NC) or miR-199a/b-3p mimics, then implanted these cells into nude mice subcutaneously, finally compared the size of their xenograft tumors and the expression of PAK4 mRNA and protein. We found that both the volume of xenograft tumors and the mRNA and protein levels of PAK4 were decreased in the group implanted with miR-199a/b-3p mimics-MGC-803 cells as compared with the group implanted with NC-MGC-803 cells (Fig. 4a and b). This results indicate that miR-199a/b-3p can suppresses GC cell proliferation through down-regulating PAK4 expression in vivo.

## Discussion

This study is first focused on miR-199a/b-3p expression and its role in regulating GC cell proliferation. Our results show that miR-199a/b-3p expression was decreased in GC and it can inhibit GC cell proliferation. We found that the expression levels of miR-199a/b-3p were significantly lower in GC tissues compared with normal adjacent tissues. Similarly, miR-199a/b-3p expression was decreased in the GC cell lines MGC-803 and SGC-7901 compared with the normal gastric mucosal cell line GES-1. In addition, we also found that the viable cell levels were significantly lower in MGC-803 and SGC-7901 cells transfected with miR-199a/b-3p mimics than in cells transfected with negative control. This results are in line with previous findings showing that miR-199a/b-3p over-expression can inhibit cell proliferation in human hepatocellular carcinoma [7] and breast cancer [14].

In further studies we sought to investigate miR-199a/b-3p antitumor mechanism. Our results show that miR-199a/b-3p inhibits GC cell proliferation via down-regulating PAK4/MEK/ERK signaling pathway. We found that miR-199a/b-3p over-expression down-regulated the mRNA and protein expression of PAK4, while PAK4 silencing suppressed MGC-803 and SGC-7901 cell proliferation. As the MEK/ERK pathway plays a critical role in PAK4-induced cell proliferation [17], we subsequently explore whether the MEK/ERK pathway was involved in miR-199a/b-3p antitumor effect by targeting PAK4 in GC. We found that both miR-199a/b-3p over-expression and PAK4 silencing down-regulated the activation of p-MEK and p-ERK in MGC-803 and SGC-7901 cells. Moreover, In our in vivo study, we also found that miR-199a/b-3p over-expression suppressed GC cell proliferation and PAK4 expression. Together, these results show that miR-199a/b-3p inhibits GC cell proliferation by the miR-199a/b-3p/PAK4/MEK/ERK axis.

Interestingly, some previous studies showed that miR-199a/b-5p was highly expressed in GC tissues compared with normal adjacent tissues and that miR-199a/b-5p functions as an oncogene in GC by targeting klotho [18–20]. Although miR-199a/b-5p and miR-199a/b-3p are the two nature forms of miR-199a/b, we identified that they not only have different miRNA sequences (Additional file 1: Table S4) but also have numerous different predicted target genes (Additional file 1: Table S5) through using bioinformatic analyses. Moreover, our finding shows that miR-199a/b-3p functions as an anti-oncogene in GC by targeting PAK4. These findings suggest that miR-199a/b-5p and miR-199a/b-3p play opposite roles in GC may by targeting different downstream genes. However, the exact mechanism underlying their opposite roles in GC needs to be further studied.

## Conclusions

In summary, our results show that miR-199a/b-3p inhibits GC cell proliferation via down-regulating PAK4/MEK/ERK signaling pathway. To our knowledge, this is the first study to demonstration that the miR-199a/b-3p/PAK4/MEK/ERK axis is involved in regulating GC cell proliferation. In addition, our finding is significant because it provides a better understanding of GC pathogenesis and offers be a novel prognostic biomarker and a potential therapeutic target for GC patients.

## Additional files

Additional file 1: Figure S1. Enrichment analysis of predicted miR-199a/b-3p targets in GCBI pathway database; **Figure S2.** MiR-199a/b-3p over-expression and PAK4 knockdown inhibited the cell proliferation ability of GC cell line 7901 in vitro as analyzed by CCK-8 assay; **Table S1.** The association of miR-199a/b-3p relative expression with the clinic-pathological characteristics in 20 GC patients; **Table S2.** Primers used in this study; **Table S3.** TargetScan prediction of miR-199a/b-3p target sites in PAK4; **Table S4.** Sequences of miR-199a/b-5p and miR-199a/b-3p; **Table S5.** Top 25 predicted targets of miR-199-3p/5p sorted by aggregate P_CT_ (DOCX 535 kb)

## Abbreviations

ERK: Extracellular signal-regulated kinase.
GC: Gastric cancer
miRNA: microRNA
PAK4: P21-Activating Kinase 4
RT-PCR: Reverse transcription-polymerase chain reaction
