# Supplementary material for: MiR-199a/b-3p inhibits gastric cancer cell proliferation via down-regulating PAK4/MEK/ERK signaling pathway
Source: BMC Cancer. 2018 Jan 5;18:34. doi: 10.1186/s12885-017-3949-2 (PMC5756398; doi:10.1186/s12885-017-3949-2)
Supplement: Supplementary file 1 — Enrichment analysis of predicted miR-199a/b-3p targets in GCBI pathway database; Figure S2. MiR-199a/b-3p over-expression and PAK4 knockdown inhibited the cell proliferation ability of GC cell line 7901 in vitro as analyzed by CCK-8 assay; Table S1. The association of miR-199a/b-3p relative expression with the clinic-pathological characteristics in 20 GC patients; Table S2. Primers used in this study; Table S3. TargetScan prediction of miR-199a/b-3p target sites in PAK4; Table S4. Sequences of miR-199a/b-5p and miR-199a/b-3p; Table S5. Top 25 predicted targets of miR-199-3p/5p sorted by aggregate PCT (DOCX 535 kb) [file 12885_2017_3949_MOESM1_ESM.docx]

**Additional file 1**

**MiR-199a/b-3p inhibits gastric cancer cell proliferation** **via down-regulating** **PAK4/MEK/ERK signaling pathway**

**Authors:** Bin Zeng, Wei Shi, Gao Tan

**Address for correspondence:** Gao Tan, Department of Gastroenterology, Nanfang Hospital, 1838 N. Guangzhou Ave., Guangzhou 510515, China, or email: tgao0316@163.com.

Bin Zeng, Department of Gastroenterology, the First Affiliated Hospital of [South China](http://www.baidu.com/link?url=w08h2iFAegA8Wlb3sdHXi9NbSsnGJPjlxpSWtp389Sm) University, Hengyang 421001, China, or email: zbss1229@163.com.


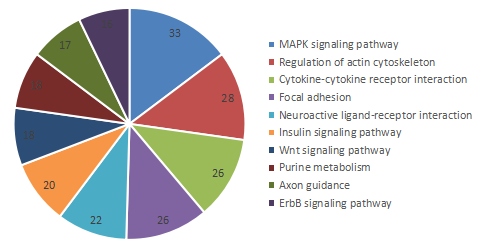


**Additional file 1: Figure S1 Enrichment analysis of predicted miR-199a/b-3p targets in GCBI pathway database**. Only the most enriched 10 cell signaling pathways of predicted miR-199a/b-3p targets in GCBI pathway database were shown.


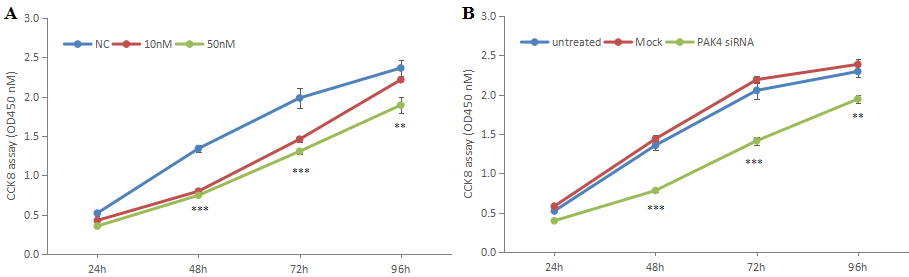


**Additional file 1: Figure S2 MiR-199a/b-3p over-expression and PAK4 knockdown inhibited the cell proliferation ability of GC cell line 7901 in vitro as analyzed by CCK-8 assay**. ***P* < 0.01; ****P* < 0.001 vs. NC or untreated groups.

**Additional file 1: Table S1 The association of miR-199a/b-3p relative expression with the clinicopathological characteristics in 20 GC patients**

| **Clinicopathological parameter** | **n** | **miR-199a/b-3p expression (cancer tissue/adjacent normal tissue)** | | ***X* ^2^** | ***P*** |
| --- | --- | --- | --- | --- | --- |
|  |  | **≤ 0.1** | **> 0.1** |  |  |
| **Gender** |  |  |  |  |  |
| Male | 12 | 8 | 4 | 0.037 | 0.848 |
| Female | 8 | 5 | 3 |  |  |
|  |  |  |  |  |  |
| **Age（year）** |  |  |  |  |  |
| <60 | 9 | 7 | 2 | 1.174 | 0.279 |
| ≧60 | 11 | 6 | 5 |  |  |
|  |  |  |  |  |  |
| **Tumor Size** |  |  |  |  |  |
| ≦5cm | 14 | 9 | 5 | 0.010 | 0.919 |
| >5cm | 6 | 4 | 2 |  |  |
|  |  |  |  |  |  |
| **Degree of differentiation** |  |  |  |  |  |
| Well | 8 | 3 | 5 | 4.432 | **0.035** |
| Moderate-Poor | 12 | 10 | 2 |  |  |

**Additional file 1: Table S2 Primers used in this study**

| Name | Primer Sequence |
| --- | --- |
| Human PAK4 F | 5'-TCCCCCTGAGCCATTGTG-3' |
| Human PAK4 R | 5'-TGACCTGTCTCCCCATCCA-3' |
| Mouse PAK4 F | 5'-CGAGTGTCCCATGAGCAGTT-3' |
| Mouse PAK4 R | 5'-TCCATCTTCTTGACGGCCAC-3' |
| Human β-actin F | 5'-AGTGTGACGTTGACATCCGT-3' |
| Human β-actin R | 5'-GCAGCTCAGTAACAGTCCGC-3' |
| Mouse β-actin F | 5'-GCAGGAGTACGATGAGTCCG-3' |
| Mouse β-actin R | 5'-ACGCAGCTCAGTAACAGTCC-3' |
| hsa-miR-199a/b-3p F | 5'-GTCACAGTAGTCTGCACAT-3' |
| hsa-miR-199a/b-3p R | 5'-GTCGTATCCAGTGCAGGGTCCGAGGTA  TTCGCACTGGATACGACTAACCA-3' |
| U6 F | 5'-CTCGCTTCGGCAGCACA-3' |
| U6 R | 5'-AACGCTTCACGAATTTGCGT-3' |
| miRNA universal reverse primer | 5'-GTGCAGGGTCCGAGGT-3' |

**Additional file 1: Table S3 TargetScan prediction of miR-199a/b-3p target sites in PAK4**

|  | **Predicted consequential pairing of target region (top) and miRNA (bottom)** | **Site type** | **P_CT_** |
| --- | --- | --- | --- |
| Position 148-155 of PAK4 3' UTR | 5'...GGUAGAUGAGACCCUACUACUGA... | 8mer | 0.87 |
|  | \|\|\| \|\|\|\|\|\|\| |  |  |
| [hsa-miR-199a-3p](http://www.mirbase.org/cgi-bin/mirna_entry.pl?acc=hsa-miR-199a-3p) | 3' AUUGGUUACACGUC--UGAUGACA |  |  |
| Position 148-155 of PAK4 3' UTR | 5'...GGUAGAUGAGACCCUACUACUGA... | 8mer | 0.87 |
|  | \|\|\| \|\|\|\|\|\|\| |  |  |
| [hsa-miR-199b-3p](http://www.mirbase.org/cgi-bin/mirna_entry.pl?acc=hsa-miR-199b-3p) | 3' AUUGGUUACACGUC--UGAUGACA |  |  |
| Position 601-607 of PAK4 3' UTR | 5'...CCCCUGCAGCAAAUGACUACUGC... | 7mer-m8 | 0.87 |
|  | \|\|\|\|\|\|\| |  |  |
| [hsa-miR-199b-3p](http://www.mirbase.org/cgi-bin/mirna_entry.pl?acc=hsa-miR-199b-3p) | 3' AUUGGUUACACGUCUGAUGACA |  |  |
| Position 601-607 of PAK4 3' UTR | 5'...CCCCUGCAGCAAAUGACUACUGC... | 7mer-m8 | 0.80 |
|  | \|\|\|\|\|\|\| |  |  |
| [hsa-miR-199a-3p](http://www.mirbase.org/cgi-bin/mirna_entry.pl?acc=hsa-miR-199a-3p) | 3' AUUGGUUACACGUCUGAUGACA |  |  |

The data was obtained from TargetScan (http://www.targetscan.org).

**P_CT_**: the probability of conserved targeting. UTR: untranslated regions.

**Additional file 1: Table S4 Sequences of miR-199a/b-5p and miR-199a/b-3p**

| Name | Sequence (5' to 3') |
| --- | --- |
| hsa-miR-199a/b-5p | 6-CCCAGUGUUCAGACUACCUGUUC-28 |
| hsa-miR-199a/b-3p | 47-ACAGUAGUCUGCACAUUGGUUA-68 |

The data was obtained from miRBase (http://www.mirbase.org)

**Additional file 1: Table S5 Top 25 predicted targets of miR-199-3p/5p sorted by aggregate P_CT_**

| **Target genes of miR-199-3p** | **Aggregate P_CT_** | **Target genes of miR-199-5p** | **Aggregate P_CT_** |
| --- | --- | --- | --- |
| [**PAK4**](http://www.ensembl.org/Homo_sapiens/Gene/Summary?g=ENSG00000130669.13) | 0.98 | [**RNF11**](http://www.ensembl.org/Homo_sapiens/Gene/Summary?g=ENSG00000123091.4) | > 0.99 |
| [**GNA12**](http://www.ensembl.org/Homo_sapiens/Gene/Summary?g=ENSG00000146535.9) | 0.98 | [**HIF1A**](http://www.ensembl.org/Homo_sapiens/Gene/Summary?g=ENSG00000100644.12) | > 0.99 |
| [**ADAMTSL3**](http://www.ensembl.org/Homo_sapiens/Gene/Summary?g=ENSG00000156218.8) | 0.98 | [**HSPA5**](http://www.ensembl.org/Homo_sapiens/Gene/Summary?g=ENSG00000044574.7) | > 0.99 |
| [**ITGB8**](http://www.ensembl.org/Homo_sapiens/Gene/Summary?g=ENSG00000105855.5) | 0.98 | [**CCNL1**](http://www.ensembl.org/Homo_sapiens/Gene/Summary?g=ENSG00000163660.7) | > 0.99 |
| [**KLHL3**](http://www.ensembl.org/Homo_sapiens/Gene/Summary?g=ENSG00000146021.10) | 0.98 | [**TRAF3**](http://www.ensembl.org/Homo_sapiens/Gene/Summary?g=ENSG00000131323.10) | > 0.99 |
| [**KIAA0319L**](http://www.ensembl.org/Homo_sapiens/Gene/Summary?g=ENSG00000142687.13) | 0.97 | [**RHEB**](http://www.ensembl.org/Homo_sapiens/Gene/Summary?g=ENSG00000106615.5) | > 0.99 |
| [**ZBTB20**](http://www.ensembl.org/Homo_sapiens/Gene/Summary?g=ENSG00000181722.11) | 0.97 | [**FSTL4**](http://www.ensembl.org/Homo_sapiens/Gene/Summary?g=ENSG00000053108.12) | > 0.99 |
| [**PIK3CB**](http://www.ensembl.org/Homo_sapiens/Gene/Summary?g=ENSG00000051382.4) | 0.97 | [**7-Mar**](http://www.ensembl.org/Homo_sapiens/Gene/Summary?g=ENSG00000136536.10) | > 0.99 |
| [**QKI**](http://www.ensembl.org/Homo_sapiens/Gene/Summary?g=ENSG00000112531.12) | 0.97 | [**MYRF**](http://www.ensembl.org/Homo_sapiens/Gene/Summary?g=ENSG00000124920.9) | > 0.99 |
| [**TAOK1**](http://www.ensembl.org/Homo_sapiens/Gene/Summary?g=ENSG00000160551.5) | 0.96 | [**CELSR1**](http://www.ensembl.org/Homo_sapiens/Gene/Summary?g=ENSG00000075275.12) | > 0.99 |
| [**NACC2**](http://www.ensembl.org/Homo_sapiens/Gene/Summary?g=ENSG00000148411.3) | 0.96 | [**MICAL3**](http://www.ensembl.org/Homo_sapiens/Gene/Summary?g=ENSG00000243156.3) | > 0.99 |
| [**ERBB4**](http://www.ensembl.org/Homo_sapiens/Gene/Summary?g=ENSG00000178568.9) | 0.95 | [**MAP3K11**](http://www.ensembl.org/Homo_sapiens/Gene/Summary?g=ENSG00000173327.3) | 0.99 |
| [**PTPN3**](http://www.ensembl.org/Homo_sapiens/Gene/Summary?g=ENSG00000070159.9) | 0.94 | [**ZBTB20**](http://www.ensembl.org/Homo_sapiens/Gene/Summary?g=ENSG00000181722.11) | 0.99 |
| [**ITGA1**](http://www.ensembl.org/Homo_sapiens/Gene/Summary?g=ENSG00000213949.4) | 0.94 | [**NCOR1**](http://www.ensembl.org/Homo_sapiens/Gene/Summary?g=ENSG00000141027.16) | 0.99 |
| [**UBE2W**](http://www.ensembl.org/Homo_sapiens/Gene/Summary?g=ENSG00000104343.15) | 0.94 | [**BCAM**](http://www.ensembl.org/Homo_sapiens/Gene/Summary?g=ENSG00000187244.6) | 0.99 |
| [**NOVA1**](http://www.ensembl.org/Homo_sapiens/Gene/Summary?g=ENSG00000139910.15) | 0.93 | [**RAD23B**](http://www.ensembl.org/Homo_sapiens/Gene/Summary?g=ENSG00000119318.8) | 0.98 |
| [**FLRT3**](http://www.ensembl.org/Homo_sapiens/Gene/Summary?g=ENSG00000125848.9) | 0.92 | [**FXR1**](http://www.ensembl.org/Homo_sapiens/Gene/Summary?g=ENSG00000114416.13) | 0.98 |
| [**CDK17**](http://www.ensembl.org/Homo_sapiens/Gene/Summary?g=ENSG00000059758.3) | 0.92 | [**ACVR2B**](http://www.ensembl.org/Homo_sapiens/Gene/Summary?g=ENSG00000114739.9) | 0.98 |
| [**NFIA**](http://www.ensembl.org/Homo_sapiens/Gene/Summary?g=ENSG00000162599.11) | 0.92 | [**NAA40**](http://www.ensembl.org/Homo_sapiens/Gene/Summary?g=ENSG00000110583.8) | 0.98 |
| [**PPP2R5E**](http://www.ensembl.org/Homo_sapiens/Gene/Summary?g=ENSG00000154001.9) | 0.91 | [**CLCN3**](http://www.ensembl.org/Homo_sapiens/Gene/Summary?g=ENSG00000109572.9) | 0.97 |
| [**NAA25**](http://www.ensembl.org/Homo_sapiens/Gene/Summary?g=ENSG00000111300.5) | 0.91 | [**YAF2**](http://www.ensembl.org/Homo_sapiens/Gene/Summary?g=ENSG00000015153.10) | 0.97 |
| [**ACVR2A**](http://www.ensembl.org/Homo_sapiens/Gene/Summary?g=ENSG00000121989.10) | 0.91 | [**STON2**](http://www.ensembl.org/Homo_sapiens/Gene/Summary?g=ENSG00000140022.5) | 0.97 |
| [**LRRC1**](http://www.ensembl.org/Homo_sapiens/Gene/Summary?g=ENSG00000137269.10) | 0.91 | [**EXOC8**](http://www.ensembl.org/Homo_sapiens/Gene/Summary?g=ENSG00000116903.6) | 0.97 |
| [**RORB**](http://www.ensembl.org/Homo_sapiens/Gene/Summary?g=ENSG00000198963.6) | 0.91 | [**LIN7C**](http://www.ensembl.org/Homo_sapiens/Gene/Summary?g=ENSG00000148943.7) | 0.96 |
| [**CCDC88A**](http://www.ensembl.org/Homo_sapiens/Gene/Summary?g=ENSG00000115355.11) | 0.90 | [**SHOC2**](http://www.ensembl.org/Homo_sapiens/Gene/Summary?g=ENSG00000108061.7) | 0.96 |

The data was obtained from TargetScan (http://www.targetscan.org).

**P_CT_**: the probability of conserved targeting.
